# Optimization of home care nurses in Canada: A scoping review

**DOI:** 10.1111/hsc.12797

**Published:** 2019-06-24

**Authors:** Rebecca Ganann, Annette Weeres, Annie Lam, Harjit Chung, Ruta Valaitis

**Affiliations:** ^1^ School of Nursing, Faculty of Health Sciences McMaster University Hamilton Ontario Canada; ^2^ Registered Practical Nurses Association of Ontario Mississauga Ontario Canada

**Keywords:** community healthcare, community nursing, home care, nursing roles, workforce development, workforce issues

## Abstract

Nurses are among the largest providers of home care services thus optimisation of this workforce can positively influence client outcomes. This scoping review maps existing Canadian literature on factors influencing the optimisation of home care nurses (HCNs). Arskey and O'Malley's five stages for scoping literature reviews were followed. Populations of interest included Registered Nurses, Registered/Licensed Practical Nurses, Registered Nursing Assistants, Advanced Practice Nurses, Nurse Practitioners and Clinical Nurse Specialists. Interventions included any nurse(s), organisational and system interventions focused on optimising home care nursing. Papers were included if published between January 1, 2002 up to May 15, 2015. The review included 127 papers, including 94 studies, 16 descriptive papers, 6 position papers, 4 discussion papers, 3 policy papers, 2 literature reviews and 2 other. Optimisation factors were categorised under seven *domains*: *Continuity of Care/Care*; *Staffing Mix and Staffing Levels; Professional Development*; *Quality Practice Environments; Intra‐professional and Inter‐professional and Inter‐sectoral Collaboration*; *Enhancing Scope of Practice*: and, *Appropriate Use of Technology*. Fragmentation and underfunding of the home care sector and resultant service cuts negatively impact optimisation. Given the fiscal climate, optimising the existing workforce is essential to support effective and efficient care delivery models. Many factors are inter‐related and have synergistic impacts (e.g., recruitment and retention, compensation and benefits, professional development supports, staffing mix and levels, workload management and the use of technology). Quality practice environments facilitate optimal practice by maximixing human resources and supporting workforce stability. Role clarity and leadership supports foster more effective interprofessional team functioning that leverages expertise and enhances patient outcomes. Results inform employers, policy makers and relevant associations regarding barriers and enablers that influence the optimisation of home care nursing in nursing, intra‐ and inter‐professional and inter‐organisational contexts.

**What is known about this topic**

- The Canadian healthcare system is underperforming given financial investments.
- Changing population demographics and system pressures to shift care from acute care to community are driving demand for home care services.
- Nurses are among the largest providers of home care; optimising the nursing workforce can enhance health system performance and positively influence outcomes for clients with increasingly complex needs.

**What this paper adds**

- Numerous inter‐related factors influence optimisation of home care nurses, who are critical members of the healthcare team.
- Results can inform other nations within similar contexts and experiencing home care sector challenges.

## 1 BACKGROUND

The Canadian Academy of Health Sciences (Nelson et al., 2000) and Fraser Institute (Barua, Hasan, & Timmermans, 2017) noted that the Canadian healthcare system is underperforming considering the financial investments that have been made; determining optimal scopes of practice for healthcare providers will be essential to inform system transformation. Furthermore, Nelson et al., assert that creativity and innovation in relation to scope of practice are required to improve Canada's healthcare system. Optimising scopes of practice in home care is particularly critical given the increasing ageing population, prevalence of patients with multiple complex conditions living at home, and demands for home care ([Link]; Home Care Ontario/Ontario Community Support Assocation Nursing Practice, 2017). With shorter hospitalisations, and increased care delivered through outpatient management, reliance on home care is growing (Canadian Institute for Health Information, 2017a). Nurses are among the largest providers of home care services; optimisation of this workforce can support optimal client outcomes while ensuring effective and appropriate use of human resources. This paper addresses a gap in synthesised literature and explores factors influencing optimisation of home care nurses (HCNs) in Canada, which will inform health system employers and policy makers struggling with similar challenges.

Home care is defined as “an array of services for people of all ages, provided in the home and community setting, that encompasses health promotion and teaching, curative intervention, end‐of‐life care, rehabilitation, support and maintenance, social adaptation and integration, and support for family caregivers” ([Link] p. 2). Across Canada, home care varies in scope of service, eligibility requirements and funding arrangements (i.e., public/private, not‐for‐profit/for profit). Mostly, home care is not covered by the Canada Health Act (Hermus, 2012) which ensures federal funding transfers to provinces for healthcare under specific conditions.

Provincially, HCNs vary in educational preparation and licensure including: Registered Nurses (RNs), Registered Practical Nurses (RPNs) and Licensed Practical Nurses (LPNs), as well as nurse practitioners (NPs). In Ontario, practical nurses are regulated as Registered Practical Nurses (RPNs). Elsewhere in Canada, they are regulated as Licensed Practical Nurses (LPNs). The term Licensed Practical Nurse (LPN) will be used hereafter. In 2015, there were over 44,000 RNs working in community health nursing (15.8% of all RNs) with 7,702 of them (3.0% of all RNs) working in direct home care (CIHI, 2017b). There were 14,159 LPNs working in community health (13.5% of all LPNs), with 3,014 working in home care (3.0% of all LPNs) (CIHI, 2017c). Although the CIHI captures data on place of work, pan‐Canadian differences in taxonomy, role definitions, and responsibilities make it difficult to accurately determine the size of the Canadian community health nursing workforce subsectors, including home care (Baumann, Underwood, et al., 2006; Underwood, Mowat, et al., 2009). Additionally, various terms are used interchangeably for nurses who work for home care agencies including home health nurse, HCN, visiting nurse and community health nurse (Baumann, Underwood, et al., 2006). For this review, HCN will be used, hereafter, to reflect diverse nurses working in the sector. Optimising the HCN workforce would enable health system efficiency and transformation (Home Care Ontario/Ontario Community Support Assocation Nursing Practice, 2017).

This scoping review maps existing Canadian literature that examined factors, which are elaborated upon below, influencing the optimisation of HCNs. For this paper, optimisation refers to strategies to support the full utilisation of nurses that strengthens patient outcomes and health system cost‐effectiveness (RNAO, ).

## 2 METHODS

Arksey and O'Malley’s (2005) five stages for scoping literature reviews were followed.

### 2.1 Stage 1: Defining the research question

The research question was defined in collaboration with nursing leaders in home and community care and other sectors on the Home Care Nursing Optimization Workgroup Advisory Sub‐Committee (henceforth named the Advisory Committee) of the Ontario Ministry of Health and Long‐term Care (including co‐authors RV, RG, AW, AL). The Advisory Committee identified seven domains considered to influence optimisation of home care nursing (Table 1) which guided the review.

**Table 1:** Domains influencing optimisation of HCNs and their definitions

Domains influencing optimisation	Definitions
1. Continuity of care and consistency of care provider	Continuity of care is “how one patient experiences care over time as coherent and linked”(Reid, McKendry, Haggerty, & Foundation, 2002). Consistency of care provider is an enabler of care continuity and refers to “…the patient's experience of a 'continuous caring relationship' with an identified healthcare professional” (Gulliford, Naithani, & Morgan, 2006) (p. 248)
2. Staffing mix and staffing levels	Staff mix is the combination of different categories of healthcare personnel employed for the provision of direct client care in the context of a nursing care delivery model (McGillis Hall et al., 2011), while staffing level refers to the number of patients per nurse and the skill mix of the staff (Royal College of Nursing, 2012)
3. Professional development to maximise nurses’ continuing competency	Professional development activities can support nurses in maintaining and continuously enhancing the knowledge, skills, attitude and judgment required to meet client needs in an evolving healthcare system (adapted from the Canadian Nurses Association [Canadian Nurses Association, 2016])
4. Quality practice environments	Quality practice environments (QPEs) maximise the health and well‐being of nurses, quality patient outcomes and organisational and system performance. Features of QPEs include benefits and compensation, job insecurity, management issues, recruitment and retention issues, safety issues, restructuring and managed competition, work‐related stress, and satisfaction (Based on RNAO's definition of a healthy work environment and six Healthy Work Environment Best Practice Guidelines (Registered Nurses' Association of Ontario, No Date)]
5. Intra‐ & Inter‐professional and Inter‐sectoral collaboration	Inter‐professional collaboration involves a variety of healthcare professionals working together to deliver quality care within and across settings, while intra‐professional collaboration involves multiple members of the same profession working collaboratively to deliver quality care within and across settings (College of Nurses of Ontario, 2014)
6. Enhancing scope of practice	Enhancing scope of practice involves implementing evidence‐based nursing roles that maximise both current scope of practice utilisation, and legislative/regulatory enhancements that expand the scope of nursing practice, to most effectively utilise the evolving knowledge, skills and competencies of the nurse to produce optimal patient/client outcomes (adapted from Primary Solutions for Primary Care [Registered Nurses' Association of Ontario, 2012b])
7. Appropriate use of technology	Appropriate use of technology includes the application of organised knowledge and skills through devices, tools, medicines, vaccines, “procedures and systems developed to solve a health problem and improve quality of lives” (from WHO's Definition of Health Technology [World Health Organization, 2016])

### 2.2 Stage 2: Identifying relevant studies

With Advisory Committee input, the inclusion/exclusion criteria and search strategy were developed and implemented. Advisory Committee engagement increased the relevance of this review for practice and policy. Inclusion/exclusion criteria are detailed below (see Table 2).

**Table 2:** Inclusion and exclusion criteria

	Population	Intervention/Exposure/Situation (where applicable)	Outcomes	Study design
Include	RNs, RPNs, LPNs, Registered Nursing Assistants (RNA), Advanced Practice Nurses (APN), Nurse Practitioners (NP) and Clinical Nurse Specialists (CNS)	Any nurse(s), organisational, and system interventions, exposures, or situations in Canada focused on optimising home care nursing	All *outcomes* relevant to the seven domains	All *study designs* including, literature reviews, primary studies, organisational quality improvement initiatives, reports, and policy and position papers
Exclude	Undergraduate education, pre‐registration nurses or nursing students, and unregulated workers	Programs or strategies delivered exclusively through hospitals, outpatient clinics located within hospital settings, primary care and public health		Conference abstracts, commentaries and editorials

Our search strategy included: electronic databases, grey literature and suggestions from Advisory Committee experts. The following databases were searched, adapting search terms according to each database's subject heading terminology and syntax requirements: MEDLINE and Pre‐MEDLINE; EMBASE; CINAHL; the Cochrane Central Register of Controlled Trials (CENTRAL); Web of Science; the National Guideline Clearinghouse and MacPLUS Federated Search. The search was conducted on January 10, 2013 and updated up to May 15, 2015, including papers from 2002 (10 years prior to the initial search) (Supporting material 1). Reference lists of included citations, key reports and organisational websites were hand‐searched (Supporting material 2).

### 2.3 Stage 3: Study Selection

Two researchers independently reviewed assigned titles and abstracts for relevance. Articles with no abstract or identified as relevant by either reviewer were retrieved for full text review. Two reviewers independently examined full texts for their assigned papers for relevance; a third reviewer resolved disagreements.

### 2.4 Stage 4: Charting the data

Data were abstracted by one reviewer and checked by a second including: year of publication; purpose; participants/population involved; type of nurse addressed; study design; research site(s); theoretical framework; provider activities; health human resources trends; optimisation barriers and facilitators by domain; outcomes and recommendations related to optimisation (Supporting material 3).

### 2.5 Stage 5: Collating, summarising and reporting the results

Stage 5 involved collating, summarising and reporting results.

## 3 RESULTS

**Figure 1:**
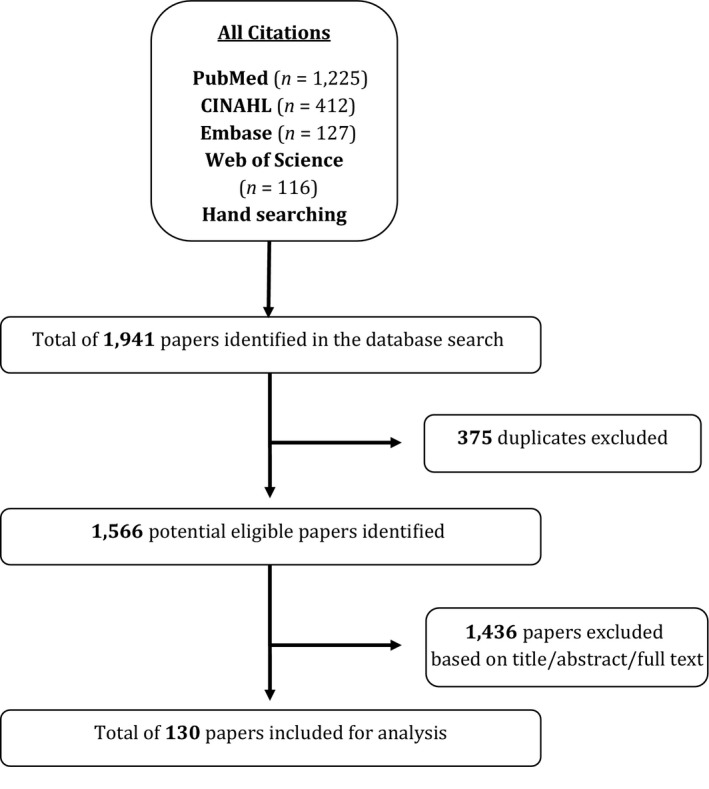
Flow chart of yield from the search

The search retrieved 1941 citations, with 1566 distinct papers following deduplication. Of these, 1764 (91%) were identified through: published literature databases, 116 (6%), grey literature searching, and 61 (3%) hand searching journals. Figure 1 presents a flowchart of literature retrieved, levels of screening and included studies. This review included 125 distinct studies, projects or reports. Two studies had two associated peer‐reviewed articles (*n* = 127 papers included). Table 3 presents studies by study design/paper type.

**Table 3:** Types of evidence included in the review (*n* = 127)

Method	Description	References
Quantitative methods (*n* = 30; 23.1%)	Cross‐sectional (*n* = 13)	Armstrong‐Stassen and Cameron (2005); Cameron et al. (2004); Caplan (2005); Denton, Zeytinoglu, and Davies (2008); Doran et al. (2007a); Doran et al. (2007b); Kaasalainen, Brazil, et al. (2011); Krueger et al. (2002); Lehoux et al. (2003); Stewart et al. (2005); Valaitis et al. (2014); Williams (2006); Zeytinoglu et al. (2009)
	Not specified (*n* = 7)	Alameddine, Laporte, Baumann, O'Brien‐Pallas, Croxford, et al. (2006); Alameddine, Laporte, Baumann, O'Brien‐Pallas, Mildon, et al. (2006); Cockerill et al. (2002); Davenport et al. (2005); Popovich et al. (2010); Shields and Wilkins (2006); Underwood, Deber, et al. (2009)
	Secondary analysis (*n* = 3)	Alameddine et al. (2014); Alameddine et al. (2009); Pitblado, Medves, and Stewart (2005)
	Randomised control trial (*n* = 3)	Markle‐Reid et al. (2013); Markle‐Reid et al. (2011); Pham, Harrison, Chen, and Carley (2012)
	Pre/post evaluation (*n* = 2)	Doran et al. (2010); Harrison et al. (2005)
	Cost analysis (*n* = 1)	Harris and Shannon (2008)
	Cohort (*n* = 1)	Harrison et al. (2011)
Mixed methods (*n* = 23; 17.7%)		Andrews et al. (2010); Canadian Homecare Association (2011); Davies et al. (2008); Denton et al. (2003); Doran et al. (2013); Ganann et al. (2010); Gifford, Davies, et al. (2013); Gifford, Graham, and Davies (2013); Gifford et al. (2014); Markle‐Reid et al. (2014); McGillis Hall et al. (2011); Mildon (2011); Mitton, O'Neil, Simpson, Hoppins, and Harcus (2007); Morin et al. (2009); Nagle and White (2013); Ogilvie et al. (2004); Pesut et al. (2015); Price et al. (2005); Shamian, Mildon, et al. (2006); Shaw, Sidhu, Kearney, Keeber, and McKay (2013); Underwood, Mowat, et al. (2009); Wagner and Gregory (2015); Zeytinoglu and Denton (2006)
Qualitative methods (*n* = 17; 13.1%)	Not specified (*n* = 5)	Abelson et al. (2004); Arnaert et al. (2009); Arnaert and Wainwright (2009); Barakat et al. (2013); Bergeron et al. (2006)
	Descriptive qualitative (*n* = 4)	Denton et al. (2014); Kaasalainen et al. (2014); Kaasalainen, Strachan, et al. (2011); Tourangeau et al. (2014)
	Ethnography (*n* = 3)	Funk and Stajduhar (2013); Giesbrecht et al. (2014); Higuchi et al. (2002)
	Grounded theory (*n* = 2)	Bediako (2002); Ploeg et al. (2014)
	Phenomenology (*n* = 1)	Marchessault et al. (2012)
	Interpretive Descriptive (*n* = 1)	Lang et al. (2009)
	Summative Evaluation (*n* = 1)	DeCicco (2008)
Descriptive paper (*n* = 16; 12.3%)		Black, Barzilay, and English (2010); Black, Barzilay, and Sheppard (2010); Canadian Homecare Association (2009); Canadian Hospice Palliative Care Association (2006); Cote and Fox (2007); Dash (2007b); Goodwin et al. (2008); Kushner et al. (2008); Lankshear et al. (2010); AETMIS (2004); Masotti et al. (2006); McWilliam et al. (2003); Meadows (2009); Meadows et al. (2014); Registered Nurses' Association of Ontario (2012b); Rivers et al. (2010)
Multiple methods (*n* = 12; 10.8%)		Baranek (2010); Baumann, Blythe, et al. (2006); Baumann et al. (2004); Baumann, Underwood, et al. (2006); Doran et al. (2012); Doran et al. (2004); Home Care Sector Study Corporation (2003a); Macleod et al. (2008); Martin Misener et al. (2008); McIsaac (2005); Underwood (2003); VON Canada (2008)
Position paper (*n* = 6; 3.8%)		Canadian Nurses Association (2013); Doran et al. (2014); Ontario Health Coalition (2011); Registered Nurses' Association of Ontario (2011, 2012a); Schofield et al. (2010)
Quality improvement paper (*n* = 5; 3.8%)		Dash (2007a); Korabek et al. (2004); Lorimer (2004); Lundrigan et al. (2010); Nasso (2006)
Discussion paper (*n* = 4; 3.1%)		Forbes and Edge (2009); Heitlinger (2003); Kulig et al. (2004); Tuggey and Lewin (2014)
Participatory action research (*n* = 4; 3.1%)		Denton et al. (2002); Longman and Gabriel (2004); Meadows and Prociuk (2012); Stevenson et al. (2008)
Policy paper (*n* = 3; 2.3%)		Canadian Healthcare Association (2009), (2011); Canadian Home Care Association (2002)
Case study (*n* = 3; 2.3%)		Denton et al. (2006); Denton et al. (2007); Morin et al. (2007)
Literature review (*n* = 1; 0.8%)		VON Canada (2005)
Scoping review (*n* = 1; 0.8%)		Macdonald et al. (2013)
Other (*n* = 2)	Think aloud method (*n* = 1)	Roberts, McLeod, Stajduhar, Webber, and Milne (2014)
	Evaluation Paper (*n* = 1)	Stacey et al. (2014)

### 3.1 Factors influencing optimisation based on each domain

Factors (*italicised*) influencing optimisation of home care and reported outcomes are presented under each domain. Table 4 lists all factors under each domain. The majority of factors under each domain are supported by multiple types of evidence which are shown in Table 4. The legend indicates the type of evidence that supports each factor. For example, in the first domain continuity of care and consistency of care provider, the factor *length of contract and job stability* was supported by a quantitative research study (QN), a mixed methods study (MM) and a qualitative research study (QL).

**Table 4:** Factors influencing optimization of home care nursing by domain (methods used in supporting evidence)

Continuity of care and consistency of care provider	1. Fragmentation in the community nursing sector (Mult.M) 2. Length of contract and job stability (QN; MM; QL) 3. Consistent scheduling and assignments (QL; QI) 4. Secure employment (QL; Mult.M; DPP) 5. Recruitment (QL; QI) 6. Stable caseloads (QI) 7. Information continuity across care transitions (QL; Mult.M)
Appropriate staff mix and staffing levels	1. Manageable workloads (QN; MM; DP; Mult.M; DPP; PAR; CS) 2. Funding models (QN; MM; QL; DP; Mult.M; CS) 3. Appropriate staff allocation (Mult.M; DPP)
Professional development	1. Orientation and access to standardized, regular, ongoing training (MM; QL; DP; Mult.M; QI; DPP) 2. Management support for professional development (QN; MM; QL; DP; Mult.M; DPP) 3. Opportunities for leadership development (MM; DP)
Quality practice environments	1. The nature of home care work (enablers and stressors) (QN; MM; QL; DP; Mult.M; QI; DPP; PAR; LR; Other) 2. Retention and recruitment (MM; QL; DP; Mult.M; DPP; CS; LR) 3. Compensation and benefits (QN; MM; QL; DP; Mult.M; QI; DPP; LR) 4. Scheduling flexibility and workload management (QN; MM; QL; Mult.M; DPP; CS) 5. Job security (QN; MM; QL; DP; Mult.M; CS) 6. System level funding (QN; QL; DP; Mult.M; DPP; PAR; CS)
Intra‐professional and, Inter‐professional and Inter‐organizational Collaboration	Intra‐professional Collaboration 1. Peer support (QN, MM, QL; DP) 2. Intra‐professional communication (QN,QL; PAR) Inter‐professional and Inter‐organizational Collaboration 1. Opportunities to interact and communicate (QN; MM; QL; DP; Mult.M; QI; DPP; PAR; Other) 2. Role clarity (QN; MM; QL; DP; Mult.M; QI; DPP) 3. Effective case management approaches (QN; MM; QL; DP; Mult.M; DPP; PAR) 4. Shared values, beliefs, and attitudes (MM; DP; Mult.M; QI; DPP; CS) 5. Adequate fiscal and human resources (Mult.M; DPP) 6. Leadership that supports collaboration and capacity building (MM, QL, Mult.M; QI)
Enhancing scope of practice	1. Changing role expectations and functions of home care nurses (QN; MM; QL; DP; Mult.M; QI; DPP; PAR; LR) 2. Organization of case management functions (QN; DP; PAR)
Appropriate technology	1. Appropriate information and communications technology use (e.g., assessment tools, electronic health records, telehealth, e‐health information) (QN; MM; QL; DP; DPP) 2. Shared electronic documentation (MM; QL; DP; DPP) 3. Staff training and education on eHealth technologies (QN; MM; QL; DP; Mult.M; DPP; PAR)

Abbreviations: CS, case study; DP, descriptive paper; DPP, discussion, policy or position paper; LR, literature review; MM, mixed‐methods; Mult.M, multi‐methods; PAR, participatory action research; QI, quality improvement paper; QL, qualitative methods; QN, quantitative methods.

#### 3.1.1 Domain: Continuity of care and consistency of care provider

Evidence for this domain is widely supported in the reviewed literature, both in the empirical literature (quantitative, qualitative, mixed and multi‐methods studies), as well in position and discussion papers. Continuity of care in home care can be challenging (Canadian Nurses Association, 2013; Doran et al., 2004, 2007a; Funk & Stajduhar, 2013) due to *fragmentation in the community nursing sector* (Underwood, 2003). *Length of contract and job stability* are positively associated with continuity and quality of care (Abelson, Gold, Woodward, O'Connor, & Hutchison, 2004; Caplan, 2005; Doran et al., 2007a; Shamian, Mildon, Goodwin, Norton, & Talosi, 2006). Organisational factors support continuity of care (Price & Lau, 2013) including: *consistent scheduling and assignments* (Denton, Brookman, Zeytinoglu, Plenderleith, & Barken, 2014; Lorimer, 2004), *secure employment* (Abelson et al., 2004; Doran et al., 2004; Home Care Sector Study Corporation, 2003a; Registered Nurses' Association of Ontario, 2011), *recruitment* (Abelson et al., 2004; Lorimer, 2004) and *stable caseloads* (Lorimer, 2004).

Continuity of care improves quality of care (Tourangeau et al., 2014), decreases confusion (VON Canada, 2008), enables therapeutic relationships (Denton et al., 2014; Tourangeau et al., 2014) and develops in‐depth provider knowledge of clients (Denton et al., 2014). It can be achieved through continuity of care provider (Baranek, 2010), better coordination and *information continuity across care transitions* (Abelson et al., 2004; Baranek, 2010; Canadian Home Care Association, 2002; Kaasalainen, Strachan, et al., 2011; Price & Lau, 2013). Both clients and nurses value consistency in care provider (Baranek, 2010; Pesut et al., 2015; Tourangeau et al., 2014).

#### 3.1.2 Domain: Appropriate staff mix and staffing levels

Factors influencing appropriate staff mix and staffing levels include *manageable workloads*, *funding models* and *appropriate staff allocation*, which can impact nursing optimisation. Evidence for this domain is also widely supported across the reviewed literature, primarily in the empirical literature (quantitative, mixed and multi‐methods studies).

Multiple papers identified *manageable workloads* influenced nursing optimisation. Insufficient time to provide quality care was an optimisation barrier (Armstrong‐Stassen & Cameron, 2005; Cockerill et al., 2002; Denton, Zeytinoglu, Davies, & Hunter, 2006; Doran et al., 2004; Kaasalainen, Brazil, et al., 2011; Markle‐Reid et al., 2014; VON Canada, 2008; Williams, 2006). Specifically, healthcare restructuring (shifting acute care delivery from hospital to community) resulted in heavier workloads and increasingly complex patients receiving home care (Denton, Zeytinoglu, & Davies, 2003; Denton et al., 2006; Williams, 2006). Increased care complexity contributed to the inability to complete complex tasks within time allotted for visits resulting in nurses working unpaid hours to provide essential care (Denton et al., 2003; Kushner, Baranek, & Dewar, 2008; VON Canada, 2008). Workload burden has resulted in nurses feeling overworked, experiencing job stress (Bediako, 2002; Denton, Zeytinoglu, Davies, & Lian, 2002; Williams, 2006), leading to decreased health, increased absenteeism and high staff turnover (Denton et al., 2003; Doran et al., 2004, 2007b). SARS contributed to increased staffing shortages (Ontario Health Coalition, 2011) and workloads (Bergeron, Cameron, Armstrong‐Stassen, & Pare, 2006), resulting in greater work‐related stress, reduced time with clients, and family life challenges (Baumann, Blythe, & Underwood, 2006; Baumann, Blythe, Underwood, & Dzuiba, 2004; Bergeron et al., 2006).

Home care *funding models* also had an important influence on nursing optimisation. Managed competition refers to a process for contracting home care services among for‐profit and not‐for‐profit organisations previously used in home care in Ontario (Abelson et al., 2004). Managed competition negatively impacted staff mixes and staffing levels. To win service contracts, home care agencies: competed for contracts through managed wages and benefits (Armstrong‐Stassen & Cameron, 2005); utilised lower costing workers (Alameddine et al., 2009; Williams, 2006); or reduced/eliminated mileage allowances (Alameddine, Laporte, Baumann, O'Brien‐Pallas, Croxford, et al., 2006; Armstrong‐Stassen & Cameron, 2005). Funding model agreements restricted optimisation of RN and RPN roles and the ability to align staffing mixes with client needs and increasing service demands (Abelson et al., 2004; Doran et al., 2012; Kushner et al., 2008; Stadnyk & Lanoix, 2011). Furthermore, multi‐year service agreements threatened the stability of home care as constraints with resultant understaffing (Abelson et al., 2004), increased workloads (Denton et al., 2006) and increased staff turnover (Abelson et al., 2004; Alameddine, Laporte, Baumann, O'Brien‐Pallas, Croxford, et al., 2006) were barriers to achieving goals and service quality (Arnaert, Seller, & Wainwright, 2009; Bradley & Nolan, 2007; Denton, Zeytinoglu, Kusch, & Davies, 2007).

Uncertainty of contract renewals and threats or loss of a contract resulted in workforce destabilisation and a "climate of fear" (Kushner et al., 2008), which negatively impacted safety, care quality care, job performance, access to professional development (Denton et al., 2006), and recruitment and retention (Abelson et al., 2004; Shamian, Mildon, et al., 2006). System impacts included increased workforce casualisation (Bediako, 2002) and organisational nursing losses to other agencies or sectors (Denton et al., 2006; Shamian, Mildon, et al., 2006).

*Appropriate Allocation of Staff* was another factor influencing nursing optimisation*.* A provincial nursing organisation's position statement recommended that organisations that employ RNs and LPNs allocate appropriate assignments based on client complexity and needs (i.e., assign RNs for complex, unstable clients with unpredictable outcomes) (Registered Nurses' Association of Ontario, 2011). Qualitative research on HCN staffing suggests that RNs and LPNs were more likely to be assigned appropriate activities if employers understood differing nursing practice roles (Baumann, Underwood, et al., 2006). Clearly written scopes of practice for RNs and LPNs could reduce role conflict, enhance role clarity and foster trust between RN and LPNs, thereby allowing RNs to focus on care management (Meadows & Prociuk, 2012).

#### 3.1.3 Domain: Professional development

The professional development domain is support by both emipirical (predominantly qualitative and mixed methods studies) and more general reviewed literature (position statements). Professional development factors that influenced the optimisation of nurses included: *orientation and access to standardised, ongoing training; management support for professional development; opportunities for leadership development;* and *access to, adequacy and availability of resources.* Professional development needs must also be addressed across the career continuum to support continuing competence. New hires who feel supported through adequate orientation (Doran et al., 2012) and preceptorship (DeCicco, 2008) are more likely to stay.

Since education typically produces generalists (Canadian Nurses Association, 2013), there is a need for *orientation and access to standardised, ongoing training* to fulfill HCN roles such as: case management, outcomes management, research, specialised technologies, program development and mental health promotion (Canadian Nurses Association, 2013; Dash, 2007b; Home Care Sector Study Corporation, 2003a; Kaasalainen et al., 2014; AETMIS, 2004; Lorimer, 2004; Markle‐Reid et al., 2014). Insufficient specialised orientation to manage increasingly complex work led to inappropriate assignments (VON Canada, 2008), workplace stress, absenteeism and burnout (Home Care Sector Study Corporation, 2003a; Zeytinoglu & Denton, 2006). It has been argued that rural nurses need to be generalists and specialists (e.g., palliative care) (Kaasalainen et al., 2014). In remote communities, a community‐specific orientation is foundational to retention (Martin Misener et al., 2008).

*Access to, adequacy and availability of resources* also influenced optimisation of HCN practice and has been shown to influence home care nursing retention (Armstrong‐Stassen & Cameron, 2005; Tourangeau et al., 2014). Many HCNs identified having inadequate learning opportunities, including time, money and access to learning resources (Underwood, Mowat, et al., 2009). Financial constraints restricted access to continuing education, subsequently affecting retention (Canadian Nurses Association, 2013; Denton et al., 2006; Home Care Sector Study Corporation, 2003b; Underwood, 2003; VON Canada, 2008). Heavy workloads and time constraints also precluded HCN's from engaging in learning opportunities (Gifford, Davies, et al., 2013; Underwood, Mowat, et al., 2009). Furthermore, travel, costs and geography created challenges to access in‐person professional development (VON Canada, 2005, 2008).

With changing population demographics and an evolving home care sector, HCNs need to develop and maintain a broad and current skill base (Bediako, 2002)^.^ Quality care is a function of nursing proficiencies arising from quality post‐licensure education, knowledge, skills and experience (Canadian Nurses Association, 2013). Many papers report benefits of ongoing professional development through in‐services or agency‐sponsored sessions to enhance skills (Canadian Nurses Association, 2013; Harris & Shannon, 2008; Harrison et al., 2011; Morin, Saint‐Laurent, Bresse, Dallaire, & Fillion, 2007; Pesut et al., 2015; Tourangeau et al., 2014). HCNs have identified gaps in knowledge and skills in: mental health, addictions, harm reduction, and stigma; chronic disease management; palliative care; and; caring for clients with increased acuity (Andrews, Morgan, & Stewart, 2010; Arnaert et al., 2009; Kaasalainen, Strachan, et al., 2011; Kushner et al., 2008; Macleod et al., 2008; Marchessault, Legault, & Martinez, 2012; Markle‐Reid et al., 2014; Schofield et al., 2010).

*Management support for professional development* was demonstrated through protected time, formal recognition and compensation, and ongoing mentor support (Meadows, 2009; VON Canada, 2008). Regular evaluation, mentorship and team supports (e.g., debriefing opportunities) were also valued (DeCicco, 2008; Higuchi, Christensen, & Terpstra, 2002; Marchessault et al., 2012; Schofield et al., 2010; Tourangeau et al., 2014; Valaitis et al., 2014; VON Canada, 2008). LPNs identified the need for coaching support to manage complex clients (Andrews et al., 2010; Doran et al., 2012).

Home care nursing leaders also require *opportunities for leadership development* to supervise and manage staff who often work in isolation (Andrews et al., 2010; Lankshear, Huckstep, Lefebre, Leiterman, & Simon, 2010). This was needed to build competence in leadership roles. Ongoing nursing leader development through distance‐learning helped increase nurses’ self‐confidence in leadership (Lankshear et al., 2010).

#### 3.1.4 Domain: Quality practice environments

Quality practice environments facilitate optimal home care practice, by maximising use of nursing human resources, supporting nurses’ satisfaction and minimising work related stress, thereby supporting HCN recruitment and retention. System level funding for home care is a significant barrier to establishing and maintaining quality practice environments. Factors influencing optimisation of Quality Practice Environments include: *the nature of home care work*; *retention and recruitment; compensation and benefits; scheduling flexibility and workload management; job security* and *system level funding.* Quality practice environments and the related factors are discussed across the reviewed literature including those using empirical methods and other discussion, policy and position papers.

The *nature of HC work* enables HCNs practice autonomy and decision‐making opportunities (Armstrong‐Stassen & Cameron, 2005; Cameron, Armstrong‐Stassen, Bergeron, & Out, 2004), which contributes to enhanced perceptions of work quality compared to nurses in acute care (Armstrong‐Stassen & Cameron, 2005; Cameron et al., 2004; McGillis Hall, Lalonde, Dales, Peterson, & Cripps, 2011) and long‐term care (Masotti, Rivoire, Rowe, Dahl, & Plain, 2006; Stacey et al., 2014). Supporting autonomy and decision‐making authority may be effective for HCN retention and optimising nursing roles (Tourangeau et al., 2014). HCN practice can be optimised through mechanisms and tools that support accountability, quality improvement, evidence‐based best practices and outcome measurement (Baranek, 2010; Popovich, Tohm, & Hurd, 2010).

The *nature of HC work* can be a source of work‐related stress (Baumann, Underwood, et al., 2006; Home Care Sector Study Corporation, 2003a; Tourangeau et al., 2014). The greatest stressor involved threats to personal safety associated with interactions with clients and families (e.g., verbal and physical abuse, aggression) (Baranek, 2010; Denton et al., 2003; Home Care Sector Study Corporation, 2003a; Kushner et al., 2008; Lang et al., 2009; Lundrigan, Hutchings, Mathews, Lynch, & Goosney, 2010; VON Canada, 2008), as well as pets (Baranek, 2010; Lang et al., 2009). Safety risks present at the point of service delivery through hazardous environments (e.g., exposure to second hand smoke, poor living conditions) (Baranek, 2010; Doran et al., 2012; Home Care Sector Study Corporation, 2003a; Lang et al., 2009), at the community level (e.g., unsafe neighbourhoods) (Doran et al., 2012; Forbes & Edge, 2009; Stevenson, McRae, & Mughal, 2008; Tourangeau et al., 2014; Underwood, Mowat, et al., 2009; VON Canada, 2008), and when travelling (e.g., weather, poor road conditions) (Canadian Nurses Association, 2013; Doran et al., 2012; Home Care Sector Study Corporation, 2003a; VON Canada, 2005, 2008). HCNs disproportionately face physical health problems, such as musculoskeletal disorders, sustained through physical strain experienced during care delivery (Cockerill et al., 2002; Denton et al., 2003; Kushner et al., 2008; Zeytinoglu & Denton, 2006).

Home care restructuring caused physical and psychological consequences of work‐related stress (Armstrong‐Stassen & Cameron, 2005; Cockerill et al., 2002; Denton et al., 2003,2002; Doran et al., 2007b; Zeytinoglu & Denton, 2006). Complexity of work under significant time constraints increased stress (Cockerill et al., 2002; Tourangeau et al., 2014; Zeytinoglu & Denton, 2006), particularly for RNs (Williams, 2006). Inadequate time for prevention activities (Cockerill et al., 2002; Tourangeau et al., 2014) and the emotional impact of palliative care work were noted as stressors (Arnaert et al., 2009; Arnaert & Wainwright, 2009; Marchessault et al., 2012). Unmanageable workloads led to stress at home infringing on personal time through unpaid duties (e.g., paperwork) (Armstrong‐Stassen & Cameron, 2005). Work‐life balance was particularly difficult to achieve for nurses working in small and remote communities (Martin Misener et al., 2008; VON Canada, 2008).

Quality practice environments influence HCNs’ *recruitment and retention*. Recruitment challenges are associated with the extensive skill set required for entry to practice (Andrews et al., 2010), little or no in‐person orientation (VON Canada, 2008), a lack of organisational support for preceptoring students (DeCicco, 2008) and limited HCN student placement opportunities (VON Canada, 2005). Recruitment facilitators include sign up bonuses, supports for orientation and skill upgrades, work flexibility, job advertisements and job fairs (Doran et al., 2012). Exposure to home care in nursing schools can influence HCN recruitment if students are better informed about roles and opportunities (Canadian Healthcare Association, 2009; Home Care Sector Study Corporation, 2003a,2003b; VON Canada, 2005). Students unprepared for home care do not consider it as a career option and new graduates entering the sector often lack home care experience (Barakat, Woolrych, Sixsmith, Kearns, & Kort, 2013; Canadian Nurses Association, 2013; Higuchi et al., 2002; Home Care Sector Study Corporation, 2003b; Macleod et al., 2008). Challenges with and high costs of recruitment underscore a need to retain the existing workforce (VON Canada, 2005). A retained workforce reduces training costs (Denton et al., 2006), promotes a stable environment (Black, Barzilay, & English, 2010; Denton et al., 2006), supports sustaining staffing requirements (Black, Barzilay, & English, 2010) and long‐term planning (Denton et al., 2006), ultimately leading to better quality care continuity for clients (Denton et al., 2006).

*Compensation and benefits,* including low wages and lack of benefits (Home Care Sector Study Corporation, 2003a; Shamian, Shainblum, & Stevens, 2006) and poor compensation for travel and visit times, negatively impacted retention (Doran et al., 2012). Home care human resources are characterised by a lack of wage parity and unsatisfactory benefits compared to hospital or long‐term care (Abelson et al., 2004; Canadian Healthcare Association, 2011; Caplan, 2005; Denton et al., 2006; Kushner et al., 2008; Ontario Health Coalition, 2011; VON Canada, 2005, 2008; Williams, 2006; Zeytinoglu & Denton, 2006), which negatively impact recruitment and retention (Alameddine et al., 2009; Caplan, 2005; Denton et al., 2003,2006; Home Care Sector Study Corporation, 2003a; Lorimer, 2004). Home care RNs were more likely to feel adequately compensated than LPNs (Home Care Sector Study Corporation, 2003a; Underwood, Mowat, et al., 2009), unionised more likely than non‐unionised (Home Care Sector Study Corporation, 2003a; Lundrigan et al., 2010), and salaried HCNs or those paid hourly more satisfied than those paid per visit (Caplan, 2005; Doran et al., 2004, 2007b; Kushner et al., 2008; VON Canada, 2008; Zeytinoglu, Denton, Davies, & Plenderleith, 2009). Other issues include lack of pay for additional work‐related activities (evening telephone calls, attending staff meetings) (Armstrong‐Stassen & Cameron, 2005; Doran et al., 2012) and a lack of pension and sick benefits (Doran et al., 2012; Home Care Sector Study Corporation, 2003a; Kushner et al., 2008). Of pocket transportation costs (e.g., car maintenance costs) and insufficient reimbursement for long distance travel to point of care were significant concerns (Alameddine et al., 2014; Armstrong‐Stassen & Cameron, 2005; Forbes & Edge, 2009; Korabek, Slauenwhite, Rosenau, & Ross, 2004)). In contrast, appropriate salaries (Baumann, Underwood, et al., 2006) and adequate reimbursement for mileage and travel time can improve working conditions and facilitate retention (Baumann, Underwood, et al., 2006; Doran et al., 2012; Home Care Sector Study Corporation, 2003a; Kushner et al., 2008).

*Scheduling flexibility and workload management* impact recruitment and retention. Flexibility in scheduling working hours was viewed as a significant benefit for HCNs (Shamian, Mildon, et al., 2006; Tourangeau et al., 2014) and supports work‐life balance (Tourangeau et al., 2014; VON Canada, 2008). HCNs also identify benefits from elect‐to‐work as control over caseload, the ability to choose desired level of work, establish therapeutic relationships with clients (Caplan, 2005), and respond to emerging client needs (Ganann et al., 2010). Workload management enabled retention and satisfaction (Armstrong‐Stassen & Cameron, 2005; Krueger et al., 2002), while time pressures to complete daily workloads had converse effects (Denton et al., 2006; Doran et al., 2007b; Tourangeau et al., 2014; Wagner & Gregory, 2015). The ability to balance work and home life can draw nurses towards home care (Shamian, Mildon, et al., 2006); however, its absence can result in turnover (Alameddine et al., 2014; Canadian Nurses Association, 2013; Caplan, 2005; Doran et al., 2004; Wagner & Gregory, 2015).

Lack of *job security* negatively affects satisfaction (Doran et al., 2004; Zeytinoglu & Denton, 2006) and retention (Armstrong‐Stassen & Cameron, 2005; Baumann et al., 2004; Caplan, 2005; Denton et al., 2003,2006; Doran et al., 2007b; Home Care Sector Study Corporation, 2003a; Kushner et al., 2008). Compared to other community health roles, HCNs experience greater employment instability (Doran et al., 2007b; Underwood, Mowat, et al., 2009). Workforce casualisation decreases the “stickiness” (retention) of HCNs in the sector (Alameddine et al., 2014), particularly for early career nurses wanting full‐time work (Doran et al., 2012). Community nurses want more job permanence, income stability and full‐time positions (Doran et al., 2004; Tourangeau et al., 2014); permanent contracts, working full‐time and salaried pay are associated with negative turnover intention (Zeytinoglu et al., 2009).

*System level funding* influences quality practice environments. Given the shifting focus from acute care to the community and home, it was estimated that by 2020, almost two‐thirds (67%) of Canadian nurses will be working in community‐based settings compared to the one‐third (30%) in 2006 (Canadian Nurses Association, 2013; Giesbrecht, Crooks, & Stajduhar, 2014). Despite rising demands, home care funding in Canada has not kept up to service demands (Auditor General of Ontario, 2015; Davenport, Rathwell, & Rosenberg, 2005; Denton et al., 2006; Higuchi et al., 2002; Schofield et al., 2010), which has contributed to barriers to optimising HCNs. HC organisations in Canada faced cost‐cutting measures (Williams, 2006) despite a growth in client numbers and complexity (Denton et al., 2002), which restricts quality of care (Higuchi et al., 2002; Home Care Sector Study Corporation, 2003a). Underfunding has also resulted in inadequate resources and equipment to provide appropriate care (Armstrong‐Stassen & Cameron, 2005; Arnaert et al., 2009; Baumann, Underwood, et al., 2006; Doran et al., 2012; Home Care Sector Study Corporation, 2003a; Martin Misener et al., 2008; McWilliam et al., 2003; Underwood, Mowat, et al., 2009; Zeytinoglu & Denton, 2006), and backups in acute care and emergency rooms (Davenport et al., 2005).

#### 3.1.5 Domain: Intra‐professional and, inter‐professional and inter‐organisational collaboration

This domain is supported by literature using a variety of methods with intra‐professional collaboation addressed primarily in emipical studies (quantitative, qualitative and mixed‐methods) and inter‐professional and inter‐organisational collaboration addressed in both the emipirical and other literature reviewed.

##### Intra‐professional Collaboration (i.e., within nursing)

P*eer support* among HCNs enabled management of heavy caseloads (Arnaert & Wainwright, 2009; Marchessault et al., 2012) and clinical decision‐making (Arnaert & Wainwright, 2009; Higuchi et al., 2002; Marchessault et al., 2012), reduced work‐related injuries, increased job satisfaction (Denton et al., 2003) and a sense of community (Wagner & Gregory, 2015). Peer support was lacking when HCNs had limited or no opportunity to discuss cases with others for example, for backup in decision‐making (Armstrong‐Stassen & Cameron, 2005; Arnaert et al., 2009; Arnaert & Wainwright, 2009; Underwood, Mowat, et al., 2009). New HCNs may face additional stressors due to limited contact with and support from peers, together with increased independence and isolation (AETMIS, 2004; Tourangeau et al., 2014).

Varying levels of nurses’ educational preparation impacted *intra‐professional communication*. HCNs experienced poor communication and a lack of cooperation from peers with different levels of educational preparation (e.g., diploma vs. degree) (Armstrong‐Stassen & Cameron, 2005; Arnaert & Wainwright, 2009). However, team leaders can help nurses with different levels of education to achieve role clarity through facilitated collaborative dialogue and rounds which helped build trust among nurses (Meadows & Prociuk, 2012). Specialist nurses working collaboratively for shared decision‐making with HCNs (e.g., palliative care) helped to develop jointly determined goals and achieve optimal outcomes (Arnaert & Wainwright, 2009).

##### Inter‐professional and inter‐organisational collaboration

Common factors influencing the HCN optimisation working in inter‐professional teams (i.e., among providers within teams and across organisations) were: *opportunities to interact and communicate*; *role clarity*; *effective case management; shared values, beliefs, and attitudes; adequate fiscal and human resources*; and *leadership that supports collaborations and joint capacity building.*

Having regular *opportunities to interact and communicate* as a team were achieved through joint meetings, consultations, workshops, debriefings, weekly inter‐professional rounds, shared decision‐making (clinical vignettes) (Arnaert et al., 2009; Baranek, 2010; Ganann et al., 2010; Higuchi et al., 2002; Home Care Sector Study Corporation, 2003a; Markle‐Reid et al., 2014; Masotti et al., 2006; Meadows & Prociuk, 2012; Stacey et al., 2014; Underwood, Mowat, et al., 2009) and conducting joint home visits (Baranek, 2010; McWilliam et al., 2003).

Inter‐professional communication challenges reported included lack of responsiveness (Armstrong‐Stassen & Cameron, 2005; Korabek et al., 2004; Markle‐Reid et al., 2014; Tourangeau et al., 2014), distance (Forbes & Edge, 2009) and lack of technology (e.g., cell phones) (Canadian Nurses Association, 2013). Challenges were also experienced in sharing information with various groups, limited time or no access to care plans, a reliance on patients to provide information from other providers, and difficulties reaching physicians to obtain orders (Baranek, 2010; Doran et al., 2012; Home Care Sector Study Corporation, 2003a; Morin et al., 2009; Price & Lau, 2013). Rural and remote nurses tend to work alone, have limited ability to collaborate with others, struggle to find consults, and need more collaboration opportunities (Andrews et al., 2010; Kaasalainen et al., 2014; Kulig, Nahachewsky, Thomlinson, Macleod, & Curran, 2004; Macleod et al., 2008). Overall, poor communication among teams led to risks to client health, clients’ poor understanding of provider roles and poor continuity of service provision (Armstrong‐Stassen & Cameron, 2005; Baranek, 2010; Canadian Healthcare Association, 2009; Shamian, Mildon, et al., 2006).

*Role clarity* was a critical factor influencing effectiveness of inter‐professional teams within and across organisations. Poor understanding of others’ roles was closely related to poor inter‐organisational communication and working in isolation (Armstrong‐Stassen & Cameron, 2005; Baranek, 2010; Baumann et al., 2004; Davenport et al., 2005; Kaasalainen, Strachan, et al., 2011; Lehoux et al., 2003; McWilliam et al., 2003; Underwood, 2003) resulting in role confusion and conflict (Andrews et al., 2010; Armstrong‐Stassen & Cameron, 2005; Caplan, 2005; Korabek et al., 2004; McWilliam et al., 2003; Registered Nurses' Association of Ontario, 2012a), and care inefficiencies (Caplan, 2005).

*Effective case management* influenced nursing optimisation. Poor coordination between sectors including private and public home care agencies, public health and non‐acute services was another barrier (Armstrong‐Stassen & Cameron, 2005; Canadian Nurses Association, 2013; Davenport et al., 2005; Meadows, Fraser, Camus, & Henderson, 2014; Registered Nurses' Association of Ontario, 2012a; Underwood, 2003). Disjointed service provision was particularly apparent during transitions in care related to a lack of information sharing (Baranek, 2010; Kaasalainen et al., 2014; Stevenson et al., 2008). Reporting to only one manager for transition care, however, effectively mitigated this challenge (Meadows et al., 2014). Other enablers of effective case management included home care managers having leadership competencies to support collaborative team work (Doran et al., 2014); having a case manager led by a nurse (Markle‐Reid, Browne, & Gafni, 2013), implementing nurse‐led health promotion interventions (Markle‐Reid et al., 2014); and using team‐based care models (Baranek, 2010). A barrier was severing the case management role from direct service provision which led to duplication, poor information sharing, and ineffective care teams (Ontario Health Coalition, 2011).

*Shared values, beliefs and attitudes* positively influenced inter‐professional work. Valuing each partner's contributions and ensuring equitable contributions of knowledge, status and authority of partners (Baumann, Underwood, et al., 2006; McWilliam et al., 2003; Morin et al., 2007; VON Canada, 2008; Wagner & Gregory, 2015) and shared philosophies or ways of working (Forbes & Edge, 2009) enabled effective collaboration. Collective belief in the benefits of collaboration contributed to increased provider satisfaction, positive client outcomes, cost savings, and nurses feeling more valued and respected as team members (Canadian Nurses Association, 2013; Korabek et al., 2004).

*Adequate fiscal and human resources* was a frequently cited factor influencing optimisation of HCN. Human resources issues influencing inter‐professional teams included: ensuring that the right person on the team is doing the right job (VON Canada, 2008); and establishing cross‐sectoral liaison positions (Canadian Home Care Association, 2002) to enhance care coordination.

*Leadership that supports collaborations and joint capacity building* was another factor influencing inter‐professional teamwork. For example, managers who encourage building new relationships; networking; community development; HCNs’ input into care and program planning; as well as demonstrate trust and recognise achievements of nurses, enabled more effective working relationships (Baumann, Underwood, et al., 2006; Ganann et al., 2010; Korabek et al., 2004; Lankshear et al., 2010; Underwood, Mowat, et al., 2009). Joint capacity building activities also can enable inter‐professional collaboration optimising HCNs, (e.g., shared training, strategic team alliances to support uptake of evidence‐based protocols) (Abelson et al., 2004; Lorimer, 2004; Nasso, 2006; Ploeg et al., 2014).

#### 3.1.6 Domain: Enhancing scope of practice

Two factors influenced nurses’ scope of practice—*changing role expectations and functions of HCNs*, and the *organisation of case management functions*. This domain is supported by both empirical and position/discussion papers within the reviewed literature.

*Changing expectations of the roles and functions of HCNs* contributed to a lack of role clarity and definition (Schofield et al., 2010); for effective community health practice, leadership needed to better understand the roles of RNs and LPNs (Ganann et al., 2010). Some administrators are unsupportive of nurses and question their work, which is juxtaposed with the need for HCNs to have the freedom to practice to full scope (Schofield et al., 2010) particularly within inter‐professional teams (McWilliam et al., 2003). With the growth in home care, nursing roles have changed to include: more care administration functions (Alameddine, Laporte, Baumann, O'Brien‐Pallas, Croxford, et al., 2006); redistribution of work (Bediako, 2002); system navigation (Caplan, 2005); collaboration with physician practices (Korabek et al., 2004); delegated tasks from the RN to the LPN (Home Care Sector Study Corporation, 2003a); and expanded roles for RNs in rural and remote regions (VON Canada, 2005). LPN roles could be maximised to include client admission assessments and leadership roles in quality improvement initiatives (Meadows & Prociuk, 2012). Barriers to HCNs working in expanded scopes of practice include a continued focus on the medical model restricting delivery of holistic care and health promotion (Underwood, 2003).

*Organisation of case management functions* further influences HCNs’ scope of practice*.* For example, RNs employed by Community Care Access Centres in Ontario took on the role of assessment and consultation, then handed off care to nursing agencies (Alameddine, Laporte, Baumann, O'Brien‐Pallas, Croxford, et al., 2006), often with LPNs to deliver care (Meadows & Prociuk, 2012). Others have identified that removing the case manager role from direct care providers has limited HCNs’ sense of autonomy and scope of practice (Kushner et al., 2008).

#### 3.1.7 Domain: Appropriate technology

This domain is supported by a mix of emipirical and discussion/policy/position papers. *Using appropriate Information and Communications Technology (ICT*) (Canadian Healthcare Association, 2009; Caplan, 2005; Doran et al., 2014; Nagle & White, 2013), screening and assessment tools (Black, Barzilay, & Sheppard, 2010; Forbes & Edge, 2009; Nagle & White, 2013), electronic health records and tele‐health (Canadian Healthcare Association, 2009), as well as providing access to information resources (Doran et al., 2010) can help optimise HCN. Commonly reported benefits of ICT use includes improved: care quality (Canadian Healthcare Association, 2009; Caplan, 2005); coordination of care (Canadian Healthcare Association, 2009; Canadian Homecare Association, 2009; Goodwin et al., 2008; Nagle & White, 2013); and, access to and exchange of information between providers (Canadian Healthcare Association, 2009; Canadian Homecare Association, 2009; Canadian Nurses Association, 2013; Denton et al., 2003; Goodwin et al., 2008; Higuchi et al., 2002). Electronic health records enabled information sharing and care continuity between HCPs, and reduced duplication of documentation and risk of errors, (Canadian Healthcare Association, 2009; Canadian Nurses Association, 2013; Caplan, 2005; Doran et al., 2014; Nagle & White, 2013).

Related to the domain inter‐organisational collaboration, *shared electronic documentation* of patient information was critical to effective collaboration (Doran et al., 2014). Use of standardised assessment tools, reporting systems, ICT, telehealth and electronic health records in home care can also lead to enhanced monitoring and management of more clients, thereby promoting efficiency (Canadian Healthcare Association, 2009) and cost savings (Barakat et al., 2013). ICT used for remote monitoring of clients enabled monitoring of chronic health conditions at a distance, therefore reducing unnecessary hospital, primary care, or home care visits (Barakat et al., 2013; Canadian Homecare Association, 2009). Furthermore, innovative technology use in rural and remote regions resulted in decreased professional isolation (Andrews et al., 2010; Forbes & Edge, 2009), and increased opportunities for distance education (Kulig et al., 2004). Failure to integrate ICT in home care can result in inefficiencies, poor inter‐professional collaboration, duplication of services and roles, difficulty contacting team members, accessing clinical support, and coordinating necessary supplies and equipment (Canadian Nurses Association, 2013).

Optimising ICT use requires *staff training and education on eHealth technologies* to increase competency and skills, as well as achieve full benefits (Barakat et al., 2013; Doran et al., 2012; Forbes & Edge, 2009; Goodwin et al., 2008; Lehoux et al., 2003; Nagle & White, 2013). Nurses have requested web‐based education to support eLearning and eTraining (Home Care Sector Study Corporation, 2003a; Longman & Gabriel, 2004); access to electronic libraries and ICT enables nurses to use evidence‐based decision‐making in practice (Black, Barzilay, & English, 2010; Gifford, Lefebre, & Davies, 2014). Managers can facilitate willingness to adapt to new technologies by: promoting sharing and learning about them (Barakat et al., 2013); ensuring adequate system speed and ease of use (Doran, Reid‐Haughian, Chilcote, & Bai, 2013); and supporting staff in technology use (Doran et al., 2013; Goodwin et al., 2008). Failure to integrate these factors creates barriers to optimising HCNs (Armstrong‐Stassen & Cameron, 2005; Barakat et al., 2013; Black, Barzilay, & Sheppard, 2010; Canadian Nurses Association, 2013; Goodwin et al., 2008; Higuchi et al., 2002; Home Care Sector Study Corporation, 2003a; Lehoux et al., 2003).

## 4 DISCUSSION

In Canada, as in Australia, the U.S., and U.K., there is a need to strengthen the home care system to meet the demands of the aging population, address the growing pressures on acute care, and manage the subsequent rising costs of healthcare (Canadian Home Care Association, The College of Family Physicians of Canada, & Canadian Nurses Association, 2016; Hartmann & Hayes, 2017; Landers et al., 2016; Morris, 2017; Palesy, Jakimowicz, Saunders, & Lewis, 2018). This scoping review explored Canadian literature published over a 13‐year span and identified 32 factors categorised under 7 domains, which were identified for their potential relevance to the optimisation of HCNs. This finding highlights the complexity of HCN optimisation. Multiple types of evidence (e.g., quantitative, qualitative, mixed methods research; descriptive papers; literature reviews; discussion papers; policy papers; and quality improvement reports) provided corroborating evidence to validate the majority of factors. A few factors such as *stable caseloads* and *fragmentation in the community nursing sector* have less evidence pointing to areas for future research.

Results can inform other nations within similar contexts and experiencing similar home care sector challenges. This scoping review has highlighted the challenge of underfunding and cuts to home care services and its impact on optimisation of HCNs. Similarly, district nurses in the UK (HCNs with a specialist qualification), who are the foundation of the home health system, have also experienced 18% funding cuts since 2010, which has led to service redesign to address growing service demands (Morris, 2017). Additionally, many Western European countries are facing HCN shortages to meet population needs (Maurits, Veer, Groenewegen, & Francke, 2017). Given this fiscal climate and the contributions of HCN, optimising the existing workforce is essential to ensure that care is not compromised.

Our review showed that the optimisation of HCNs must be considered in the context of interprofessional teams. Role clarity and leadership that supports collaborative work are important supporting factors for high‐functioning teams. Internationally, nurses are among the core providers of seniors’ care working with personal care workers and rehabilitation professionals (2014 & Michael Smith Foundation for Health Research, 2^2014^; Morris, 2017). Similarly, Canada's recently released action plan for home care states: “A key pillar of integrated community‐based care is leadership from physicians, nurses and other healthcare providers, working to their full scope of practice, within fully functioning teams” (Canadian Home Care Association, 2016) (p.11). As interprofessional teams in healthcare have become more commonplace with nurses as integral members, it is critical that each team member's role is optimised and expertise leveraged to collaboratively support patient care (CIHI, 2017a).

As in our scoping review, the UK and European nations have reported that HCN are experiencing burnout related to the heavy workloads and emotional demands of home care nursing which is exacerbated by a lack of recruitment to home care nursing and an ageing workforce (Morris, 2017; Vander Elst et al., 2016). A Belgian study found that task autonomy, social support and opportunities for learning could buffer workplace stresses (Vander Elst et al., 2016). A recently published Canadian study (Tourangeau, Patterson, Saari, Thomson, & Cranley, 2017) confirmed our findings that HCN retention was related to a number of modifiable factors including: income stability, meaningfulness of work, continuity of care, positive relationships with supervisors, work‐life balance, and satisfaction with salary and benefits. One factor related to retention in this study that was not revealed in our review included nurses’ perceptions of the quality of care provided by their organisation (Tourangeau et al., 2017).

Krietzer and colleagues argue that dissatisfaction in the US nursing workforce is related to bureaucratic structures, poor working conditions, and a loss of autonomy which has led to shortages in the workforce (Kreitzer, Monsen, Nandram, & De Blok, 2015). In contrast, an innovative self‐directed nursing team model of care—Buurtzorg—developed in the Netherlands has shown to increase nursing satisfaction and a sense of autonomy over patient care, particularly for nursing assistants and bachelor's degree prepared nurses (Maurits et al., 2017). These autonomous nurse‐led teams have been shown to spend little time on administration by using computerised systems, make local connections, and support continuity in care (Dharamshi, 2014; Sheldon, 2017). Other characteristics of the model include: nursing engagement in developing creative solutions to problems, simplified billing, financial stability, low overhead, web‐based communities of practice, and administrative management (Kreitzer et al., 2015). Such innovative models in home care need to be explored more fully in light of growing care demands and fiscal realities as their potential to fully optimise HCN.

Of the 127 including papers, most conducted descriptive studies or program evaluations using qualitative (*n* = 30), qualitative (*n* = 17) or mixed methods approaches (*n* = 23), while three papers involved an experimental study and one applied an uncontrolled quasi‐experimental study. This points to the need for rigorous experimental studies to better understand what and how interventions can support optimisation of HCNs while ensuring positive patient outcomes. Given the complexity inherent in the optimisation of HCNs, future pragmatic trials are recommended. The strength of pragmatic trials is that they are implemented in real world conditions allowing for their results to be applied in routine practice settings (Patsopoulos, 2011).

A limitation of this scoping review is that many of the primary sources lacked specifics to identify roles, functions, and educational preparation of the nurses. As such, we were unable to map the literature by sub‐populations of nurses. This scoping review did not assess the scientific rigor of research studies, however it followed all recommended steps of a scoping review (Arksey & O'Malley, 2005) and maps over 20 years of home care nursing literature of Canada.

## 5 CONCLUSIONS

The results from this review of Canadian literature highlights that a broad range of complex and interrelating factors influence the optimisation of the HCN workforce. These results can inform policy makers, home care employers, managers and service providers within Canada and beyond on strategies to optimise home care nursing, since other nations report similar challenges in meeting demands for HCN services. It is critical to ensure that the HCN workforce works to full scope, ensuring appropriate staffing and skills mix working in teams, role clarity and leadership support, which are supported by technology and quality practice environments in order to meet the complex needs of patients needing nursing care.
