# Supplementary material for: Optimization of home care nurses in Canada: A scoping review
Source: Health Soc Care Community. 2019 Jun 24;27(5):e604–21. doi: 10.1111/hsc.12797 (PMC6851676; doi:10.1111/hsc.12797)
Supplement: Supplementary file 1 [file HSC-27-e604-s001.docx]

**Supporting material file 1: Electronic database search strategy**

Databases searched:

- PubMed
- Embase
- CINAHL
- Web of Science search

**Search terms:**

(((((home AND (health OR care)) OR visiting OR rural OR remote OR community)) AND (nurse OR nursing)) AND (((nursing OR health) AND human resources) OR system integration OR service coordination OR staff mix OR staffing OR organizational support OR professional development OR ((continued OR continuing) AND education) OR evidence-based OR evidence-informed OR evidence based OR evidence informed OR scope of practice OR quality health care OR (tele-nursing OR tele-health OR telehealth) OR technology OR stickiness OR magnet OR retention OR recruitment OR ((nurse OR nursing) AND engagement) OR job satisfaction OR competence OR (practice setting OR practice environment OR workplace OR healthy work environment) OR collaboration OR partnership OR intersectoral OR inter-sectoral OR ((inter OR intra) AND (professional OR disciplinary)))) AND Canada

**Filters:**

Dates: Jan. 1, 2002 to Dec. 31, 2012; updated to include Jan. 1, 2013 to April 30, 2015

**Language:** English
