# Supplementary material for: Optimization of home care nurses in Canada: A scoping review
Source: Health Soc Care Community. 2019 Jun 24;27(5):e604–21. doi: 10.1111/hsc.12797 (PMC6851676; doi:10.1111/hsc.12797)
Supplement: Supplementary file 2 [file HSC-27-e604-s002.docx]

**Supporting material 2: Key reports and websites hand searched**

**Key reports:**

- Vision 2020, Community Health Nurses of Canada
- CNA report
- RNAO ECCO report: Enhancing Community Care for Ontarians – White Paper <http://www.homecareontario.ca/documanager/files/news/rnao_ecco_white_paper_final.pdf>
- RNAO Primary care report
- Nursing Secretariat reports via CJNL – Nursing Human Resource demonstration project

<http://www.longwoods.com/product/download/code/21732>

- NHSRU Home Health Nurses in Ontario, 1999-2009
- Home Care in Canada: From the Margins to the Mainstream, 2009, Canadian Healthcare Association
- Mildon, B. (2011). The Concept of Home Care Nursing Workload. Unpublished doctoral dissertation, University of Toronto. <https://tspace.library.utoronto.ca/bitstream/1807/29508/1/Mildon_Barbara_L_201106_PhD_thesis.pdf>
- <http://www.nhsru.com/publications/building-and-sustaining-a-quality-nursing-workforce-in-home-and-community-care-march-2012-interim-report>
- <http://www.nhsru.com/category/publications/community-health-home-care>

# Valaitis et al. (2014). Community health nurses’ learning needs in relation to the Canadian community health nursing standards of practice: results from a Canadian survey. *BMC Nursing*. 13:31

- Berry, L., and Curry, P. (2012) *Nursing Workload and Patient Care* via Canadian Federation of Nurses Unions website.

**Websites:**

<http://www.cdnhomecare.ca/>

<http://www.cha.ca/>

<http://www.wecare.ca/>

<http://www.nursesunions.ca/>

<http://www.cna-aiic.ca/en/>

<http://www.parl.gc.ca/Content/LOP/ResearchPublications/2012-03-e.pdf>

<http://www.homecareontario.ca/public/>

<http://www.ocsa.on.ca/>

<http://www.ona.org/>

<http://www.paramed.com/>

<http://www.bayshore.ca/>

http://www.von.ca/en/resources

<http://saintelizabeth.com/>

<http://www.ccac-ont.ca/>

<http://www.lhins.on.ca/home.aspx?LangType=4105>

<http://www.chnig.org/>

<http://www.chnc.ca/>

<http://www.rpnao.org/>

<http://rnao.ca/>
