# Supplementary material for: Optimization of home care nurses in Canada: A scoping review
Source: Health Soc Care Community. 2019 Jun 24;27(5):e604–21. doi: 10.1111/hsc.12797 (PMC6851676; doi:10.1111/hsc.12797)
Supplement: Supplementary file 3 [file HSC-27-e604-s003.doc]

| ***Ref ID:*** | |
| --- | --- |
| ***Author:***  ***Publication Date:***  ***Extraction done by:*** | |
| Reviewer General Comments/Notes: | |
| Purpose of paper/ study |  |
| Participants (involved in the study) or population of interest (if not study) |  |
| Nurses addressed in paper | CNS Clinical Nurse Specialist (Advanced Practice)  Nurse practitioners  Registered nurses  Licensed practical nurses/ Registered Practical Nurses  nursing students  Other ___________  Unclear nurses are implied in population |
| Methods (for studies - e.g. RCT, Systematic review, Mixed methods) |  |
| Site where (context: i.e. province, urban, rural) |  |
| Theoretical framework (if applicable) |  |
| Activities of disciplines / organizations involved (who is involved, what did they do) |  |
| **Results / Outcomes** | |
| Trends and Stats on HHR in HC Nursing |  |
| - Continuity of Care and Consistency of Care Provider   Barriers:  Facilitators: |  |
| - Staff mix and staffing levels   Barriers:  Facilitators: |  |
| - Professional development to maximize nurses’ continuing competency   Barriers:  Facilitators: |  |
| - Quality practice environments   Barriers:  Facilitators: |  |
| - Intra-interprofessional collaboration and/or inter-sectoral collaboration   Barriers:  Facilitators: |  |
| - Technology (use of technologies to optimize HC nurses’ work)   Barriers:  Facilitators: |  |
| - Expanding HC nursing roles |  |
| Other Results |  |
| Comments/Theme Extraction: |  |
| Recommendations from authors for Optimization of Home Care Nursing |  |
